# High Expression and Purification of Amino-Terminal Fragment of Human Amyloid Precursor Protein in *Pichia pastoris* and Partial Analysis of Its Properties

**DOI:** 10.1155/2013/836429

**Published:** 2013-11-07

**Authors:** Wei Li, Xiang Gao, Junle Ren, Ting An, Yan Liu

**Affiliations:** School of Life Science, Southwest University, Chongqing 400715, China

## Abstract

The cleaved amino-terminal fragment of human amyloid precursor protein (N-APP) binds death receptor 6 (DR6) and triggers a caspase-dependent self-destruction process, which was suggested to contribute to Alzheimer's disease. To investigate the N-APP-DR6-induced degeneration pathway at the molecular level, obtaining abundant and purified N-APP is fundamental and critical. The recombinant N-APP has been produced in mammalian expression system. However, the cost and yield disadvantages of mammalian expression system make it less ideal for protein mass production. Here, we successfully expressed and purified recombinant N-terminal 18-285 amino acid residues of human amyloid precursor protein from the methylotrophic yeast *Pichia pastoris* with a high yield of 50 mg/L. Flow cytometry indicated the purified N-APP-induced obvious apoptosis of human neuroblastoma SHEP cells.

## 1. Introduction

Alzheimer's disease (AD) is the most prevalent degenerative disease of the central nervous system. The amyloid precursor protein (APP) has been extensively studied because of its association with the pathogenesis of AD [1]. APP is expressed in adult brain and upregulated in damaged axons [2]. Death receptor 6 (DR6, also known as TNFRSF21) is also highly expressed in adult brain. Given their findings, it is reasonable to assume that the APP-death-receptor mechanism might contribute to adult plasticity or to neurodegeneration after injury or in disease. Interestingly, DR6 is upregulated in injured neurons [3], raising the question as to whether overexpressed DR6 in neurons can trigger ligand-independent degeneration, as reported for p75 neurotrophin receptor (p75NTR) [4]. Given the genetic evidence linking APP and its cleavage to AD, it was proposed that signalling of APP via DR6 (and possibly p75NTR) may in particular contribute to the initiation or progression of AD, either alone or in combination with other proposed APP-dependent mechanisms, such as amyloid *β*-peptide (A*β*) toxicity [5, 6] or effects of the APP intracellular domain [7]. Of note, previous studies showed immunoreactivity for the APP N-terminus associated with Alzheimer's plaques [8, 9], the APP-death-receptor mechanism could support a model in which trophic-factor (TF) deprivation triggers the cleavage of surface APP by *β*-secretase, releasing the soluble ectodomain of APP (sAPP*β*), which is further cleaved by an unknown mechanism to release the amino-terminal fragment of human amyloid precursor protein (N-APP). This then binds DR6 to trigger degeneration through caspase 6 in axons and caspase 3 in cell bodies [10].

Thus, further study is required to determine the full implications of the APP-death-receptor mechanism in development, adult physiology, and disease. Nonetheless, the results already tied APP to a new mechanism for neuronal self-destruction in development and suggested that the APP ectodomain, acting via DR6 and caspase 6, contributes to the pathophysiology of AD.

 The availability of pure APP isoforms is essential for studying their processing and biological function. Various expression systems have been used to produce biologically active APP isoforms including bacteria (APP695) [11, 12] and baculovirus (APP695, −751, and −770) [13, 14]. Recently, APP695, APP751, and the Kunitz-type protease inhibitor (KPI) domain of an APP homolog cloned from human placenta have been expressed in the yeast *Saccharomyces cerevisiae* [15]. The methylotrophic yeast *Pichia pastoris* has been used to produce the ectodomains of APP695, APP751, and APP770 [16, 17] and the KPI domain of APP [18, 19]. Although recombinant N-APP (1-286) could have been produced in mammalian cell line system, mass production of N-APP is the prerequisite for its wide application it needs high cost. The high efficient production of N-APP, with large quantity and low cost, is in urgent need for both further fundamental investigation and potential application. The *P. pastoris *expression system is being used successfully and extensively for the production of various recombinant heterologous proteins. *P. pastoris* is also a eukaryote and thereby provides the potential for producing soluble, correctly folded recombinant proteins that have undergone all the co- and post-translational modifications required for functionality. Recently, the genomic sequence of the GS115 strain of *P. pastoris* has been presented [20].

 Here, we report that N-APP (18-285) was highly expressed in the methylotrophic yeast *P. pastoris* and establish the purification procedure. It was detected that the apoptosis rate of human neuroblastoma SHEP cells dealt with different concentrations of N-APP, which proved that N-APP had induced apoptosis on SHEP cells. Thus, a recombinant eukaryotic yeast strain expressing N-APP was constructed successfully.

## 2. Materials and Methods

### 2.1. Materials

Plasmid pCMV-SPORT6, a vector with the APP gene (GenBank Accession No. BC065529) was purchased from Seajet Scientific Inc. (Beijing, China). *Escherichia coli *Top10*F*′ was maintained by our lab. *P. pastoris* wild-type strain GS115 and the expression vector pPIC9K plasmid were purchased from Invitrogen Life Technologies (Carlsbad, CA, USA). All chromatographic media (Q Sepharose fast flow, Sephacryl S-200 high resolution gel filtration) and HiTrap desalting column were obtained from GE Healthcare (Piscataway, NJ, USA). Polymerase chain reaction (PCR) products used for cloning were confirmed by sequencing at Invitrogen (Shanghai, China). Protein molecular weight markers were from New England Biolabs (Ipswich, MA, USA). Mouse anti-His monoclonal antibody and secondary antibody horseradish peroxidase- (HRP-) conjugated goat anti-mouse immunoglobulin G (IgG) used for western was obtained from the Antibody Research Center (Shanghai Institute of Biochemistry and Cellular Biology, Chinese Academy of Sciences) and Santa Cruz Biotechnology (Santa Cruz, CA, USA), respectively. All other chemical reagents were of analytical grade.

### 2.2. Construction of the Expression Vector

DNA encoding the mature N-terminal lysine (amino acid 18) to the *α*-secretase site of APP285 (amino acid 285) was prepared by PCR using the 5′ forward primer 5′CCGCTCGAGAAAAGACTGGAGGTACCCACTGATGGTAATG3′ and the 3′ reverse primer 5′GGAATTCCTTATTAATGATGATGATGATGATGGGATCCTCTTGGAACCAACTCTTCCACAGACTCTGTGGTGGTG3′, pCMV-SPORT6 as the template. These oligonucleotides include an *Xho* I and an *Eco*R I site for cloning into the *Xho* I and* Eco*R I site of the *P. pastoris* expression vector pPIC9K. The 3′ reverse primer contained double stop codon. In order to express the native N-terminus of APP, an *Xho* I restriction site was introduced to allow in-frame cloning into the *α*-factor secretion signal of pPIC9K expression vector, and a nucleotide sequence encoding the KEX2 cleavage site was placed ahead of N-APP (18-285). At the C-terminus, the stop codon and a nucleotide sequence encoding the thrombin cleavage site were introduced along with an *Eco*R I restriction site (Table 1). The PCR product was digested with *Xho* I and *Eco*R I and cloned into the same sites of pPIC9K. The recombinant plasmid pPIC9K-N-APP (18-285) was transformed into competent *E. coli* Top10*F*′, and the fragment was validated by restriction enzyme digestion with *Xho* I and *Eco*R I and confirmed by DNA sequencing.

### 2.3. Transformation of *P. pastoris* and Selection of Transformants

*Sac *I was used to linearize 5–10 *μ*g pPIC9K-N-APP plasmid DNA, and linear DNA was then transformed into the competent* P. pastoris* GS115 cells using a Gene Pulser system (Bio-Rad; conditions used: 1.5 kV, 200 Ω, 25 *μ*F, and 4.5 ms). pPIC9K blank plasmid vector alone was also linearized and transformed into *P. pastoris* GS115 cells as a negative control. After initial selection of histidine utilization plus (His^+^) transformants on regeneration dextrose medium (RDB) plates which did not add histidine, they were spread on yeast extract peptone dextrose medium (YPD)-geneticin plates with 3 mg/mL. All geneticin-resistant colonies were plated in duplicate onto either minimal methanol medium (MM) or minimal dextrose medium (MD) plates to characterize the methanol-utilizing phenotype. The methanol utilization plus (Mut^+^) phenotype strains were obtained from MM plates, and the inserts were verified by colony PCR.

### 2.4. Expression and Purification of Recombinant Protein in *P. Pastoris*

Positive transformants were tested for their ability to secrete N-APP into the cell culture supernatants using the method according to the Invitrogen manual. The high yield strain was inoculated into a flask containing 100 mL buffered glycerol-complex medium (BMGY). Shaken at 30°C, 250 rpm for 48 h, the culture was centrifuged at 4°C, 3000 g for 5 min. The collected cells were resuspended in 200 mL buffered methanol-complex medium (BMMY) and allowed to grow at 30°C for 72 h. Methanol was added at the first 24 h to a final concentration of 1.0% to maintain induction. The culture was centrifuged at 6000 rpm for 20 min and then analyzed by sodium dodecyl sulfate polyacrylamide gel electrophoresis (SDS-PAGE). The filtered expressed supernatant was desalted for buffer exchange on HiTrap desalting column; exchanging buffer was lysis buffer (20 mM imidazole, 5 mM EDTA, pH 6.0). The sample was then loaded onto a Q-Sepharose fast flow column (Q-Sepharose FF column, 1.6 × 25 cm, flow rate 10 mL/min). The column was preequilibrated with buffer A (20 mM imidazole, 50 mM NaCl, 5 mM EDTA, pH 6.0) then washed with buffer A and the bound protein was eluted with buffer B (20 mM Tris-HCl, 500 mM NaCl, 5 mM EDTA, pH 7.4), and the eluting proteins were collected (50 mL). The eluted fractions were concentrated by centrifugal filter devices Amicon Ultra-15 (Millipore), and further purified using Sephacryl S-200 high resolution which was equilibrated and eluted with 20 mM Tris-HCl, 150 mM NaCl, pH 8.0. The purified protein was analyzed by 12% SDS-PAGE.

### 2.5. Gel Electrophoresis and Western Blotting

Samples were separated by SDS-PAGE and electrophoretically transferred onto nitrocellulose (Millipore). Primary antibodies were detected with mouse anti-His monoclonal antibody secondary antibody using HRP-conjugated goat anti-mouse, the specific protein bands were incubated with diaminobenzidine (DAB) kit (Amersham Pharmacia Biotech).

### 2.6. Stability of the Purified Protein

After being purified by Q-Sepharose FF column, the recombinant proteins were placed at 4°C and 20°C for 4 d, respectively, then analyzed by SDS-PAGE.

### 2.7. Effect of Recombinant N-APP on SHEP Cells *In Vitro* Analyzed by Flow Cytometry

To explore the effect of the human recombinant N-APP protein on neurocyte, we observed the influence of N-APP on the apoptosis of SHEP. The logarithmic phase cells were processed with different concentrations of N-APP protein followed by morphological evaluation. 

### 2.8. Statistical Analysis

Data were analyzed using Excel (version 2007) and represented as mean ± standard deviation (SD) between replicates. At least two independent experiments were performed for each analysis, and the number of replicates (*n*) for each experiment is indicated. Statistical significance was evaluated by Student's *t*-test; *P* < 0.05 was considered as significant; *P* < 0.01 was considered as statistically extremely significant. *P* values are indicated by asterisks in the graphs (*P < 0.05; **P < 0.01).

## 3. Results

### 3.1. Construction and Identification of Recombinant Expression Vector

**Figure 1:**
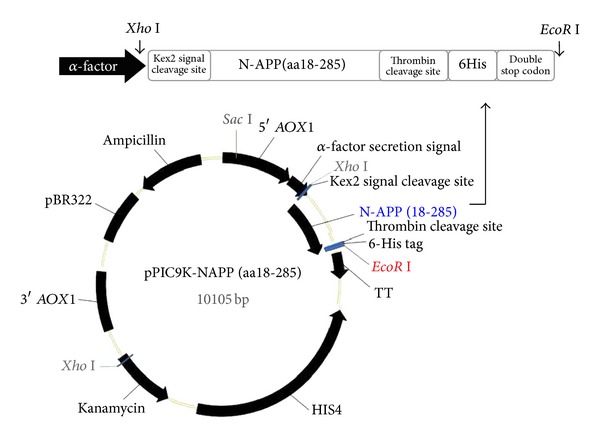
Design of recombinant plasmid pPIC9K-N-APP (18-285).

The *Xho *I and* Eco*R I sites were used to insert the DNA fragment encoding the human N-APP (18-285) in the *P. pastoris* expression vector pPIC9K which contains an *S. cerevisiae *alpha factor leader and an alcohol oxidase 1 (*AOX1*) promoter (Figure 1). There was a site-specific recognition sequence of thrombin between 6His-tag and N-APP (18-285) gene fragment for the removal of His tag. The constructed structure of the expression vector was confirmed by restriction enzyme digestion and DNA sequencing (data not shown).

### 3.2. Expression and Western Blotting

**Figure 2:**
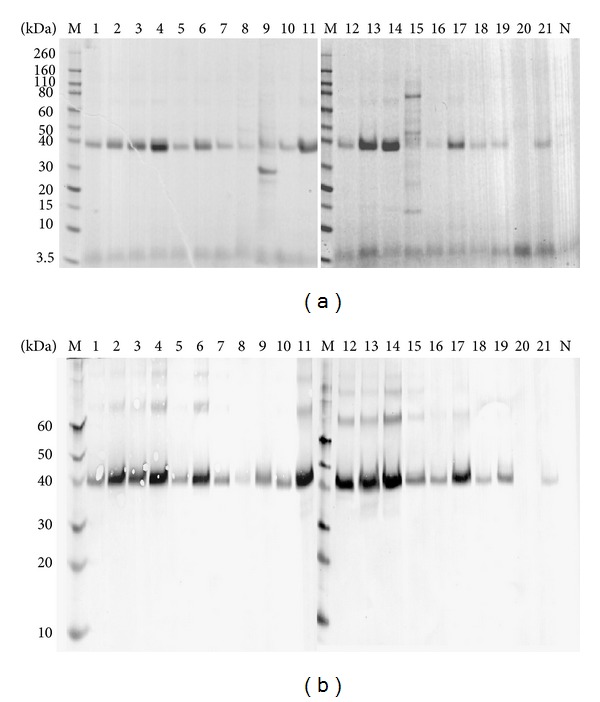
(a) SDS-PAGE screening of high expression recombinant strain. M: molecular weight protein marker; N: negative control GS115/pPIC9K; lanes 1–21: expressed supernatants GS115/pPIC9K-N-APP; (b) Western blot analysis of recombinant proteins. M: prestained protein molecular weight marker; lanes 1–21: expressed supernatants GS115/pPIC9K-N-APP; N: negative control GS151/pPIC9K.

The positive colonies were cultured and induced to screen high-expression recombinant strains. SDS-PAGE analysis showed that N-APP (18-285) was successfully expressed in the* P. pastoris* GS115 transformant. The molecular weight of the protein was approximately 38 kDa as determined by SDS-PAGE (Figure 2(a)). Western blotting analysis showed that the protein was recognized specifically by mouse anti-His antibody demonstrating that the expressed heterogeneous protein was recombinant N-APP (18-285) (Figure 2(b)).

### 3.3. Purification of N-APP

**Figure 3:**
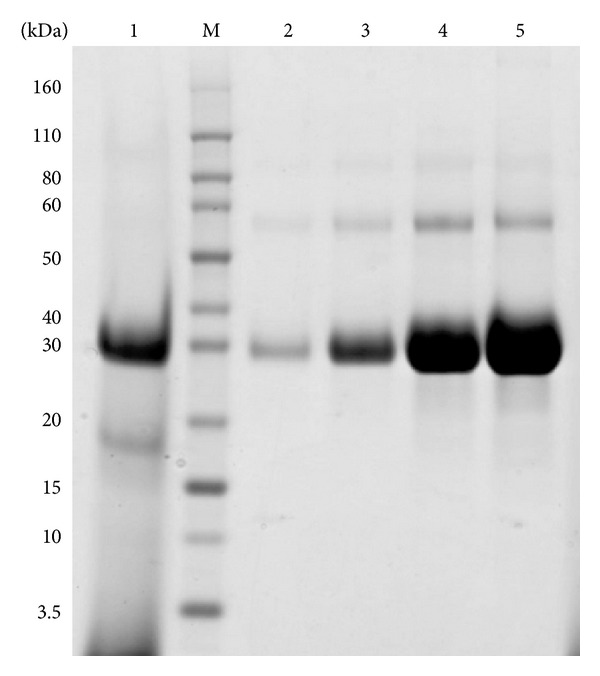
Purified recombinant N-APP by Q Sepharose fast flow chromatography. M: protein marker; lane 1: culture supernatant; lanes 2–5: purified N-APP from different tubes after ion exchangers.

**Figure 4:**
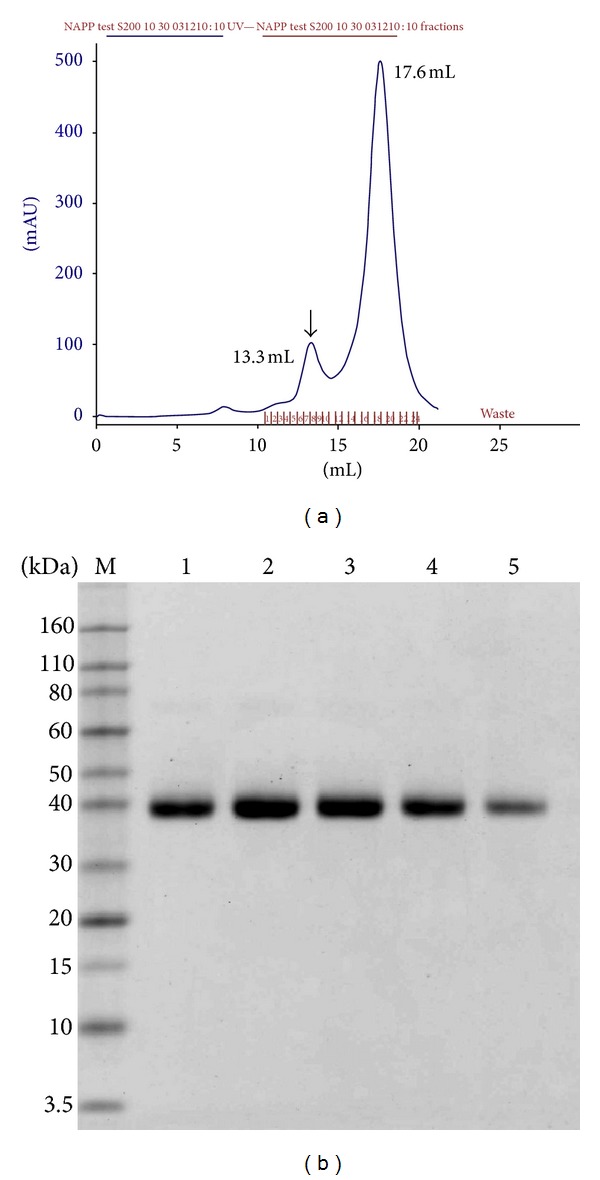
Purified recombinant N-APP by Sephacryl S-200 high resolution chromatography. (a) Profile of gel filtration of the fractions from the Q Sepharose fast flow chromatography. The arrow indicates the purified protein eluting peak (13.3 mL) which corresponds to the 6th, 7th, 8th, 9th, and 10th collection tube; (b) SDS-PAGE analysis of the purified N-APP after gel filtration. M: protein marker; lanes 6–10: the eluting fraction which corresponds to the 6th, 7th, 8th, 9th, and 10th collection tube.

The culture supernatant was primarily purified by a Q Sepharose FF column (Figure 3). The fractions containing the target protein were collected and concentrated by centrifugal filter devices. N-APP was further purified with Sephacryl S-200 high resolution column (Figure 4(a)). Following this process, the purity of N-APP was >95%, as revealed by SDS-PAGE (Figure 4(b)). The purified protein was estimated using the bicinchoninic acid (BCA) assay, and its yield was about 50 mg/L (Table 2).

### 3.4. Stability of the Purified Protein

**Figure 5:**
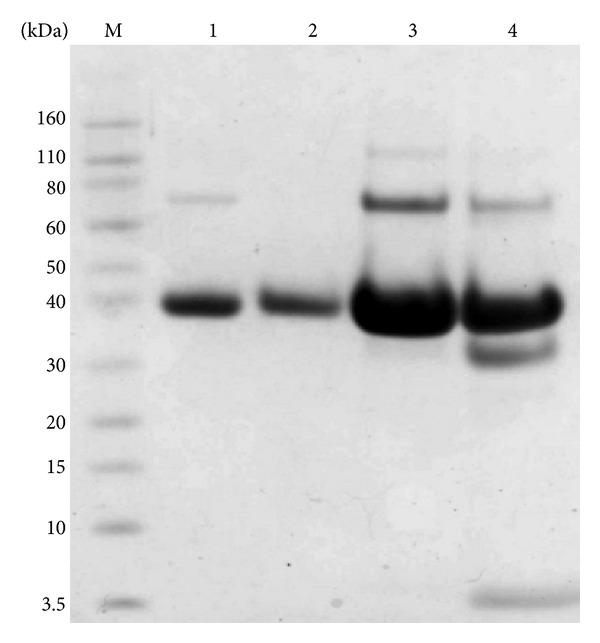
SDS-PAGE analysis of stability of the purified protein. M: protein marker; lane 1, 2: purified protein by Q Sepharose fast flow chromatography; lane 3: the purified protein by Q Sepharose fast flow chromatography which was incubated at 4°C for 4 d; lane 4: the purified protein by Q Sepharose fast flow chromatography which was incubated at 20°C for 4 d.

When the recombinant proteins were purified using Q-Sepharose FF column, the bound protein was eluted with buffer B (20 mM Tris-HCl, 500 M NaCl, 5 mM EDTA, pH 7.4). At pH 7.4, the temperatures (at 4°C and 20°C) for stability of the recombinant proteins were studied. We put the recombinant proteins under 4°C and 20°C for 4 d, respectively. SDS-PAGE electrophoretograms of the recombinant proteins showed that the proteins were obviously not degraded (Figure 5). 

### 3.5. Effect of Recombinant N-APP on SHEP Cells *In Vitro*

**Figure 6:**
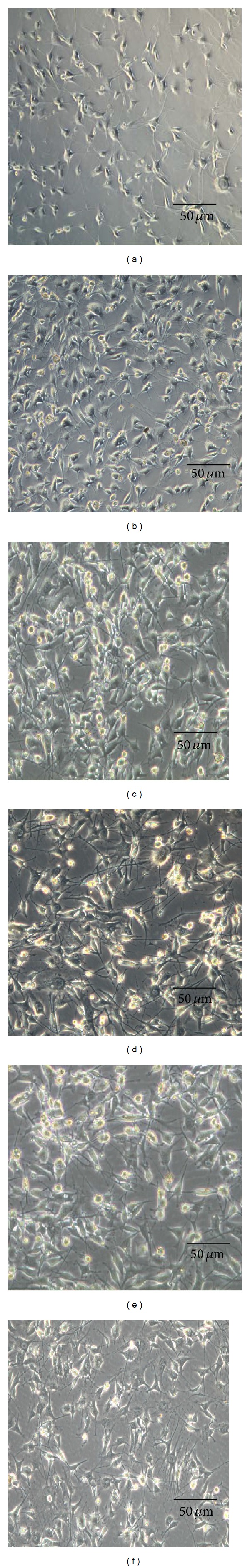
Effect of N-APP on SHEP cells cultured 24 h (200x). (a) Control; (b)–(f): N-APP at different concentrations of 2.5, 5, 10, 20, and 40 *μ*g/mL.

Recombinant N-APP changes the morphology of SHEP cells. Proliferating SHEP cells were treated with N-APP at different concentrations of 0, 2.5, 5, 10, 20, and 40 *μ*g/mL. Pictures (Figure 6) were taken 24 h after treatment. In the controls, the cells showed intact cell morphology, whereas N-APP-treated cells appeared rounded and floating and showed membrane blebbing characteristic of apoptotic cells.

**Figure 7:**
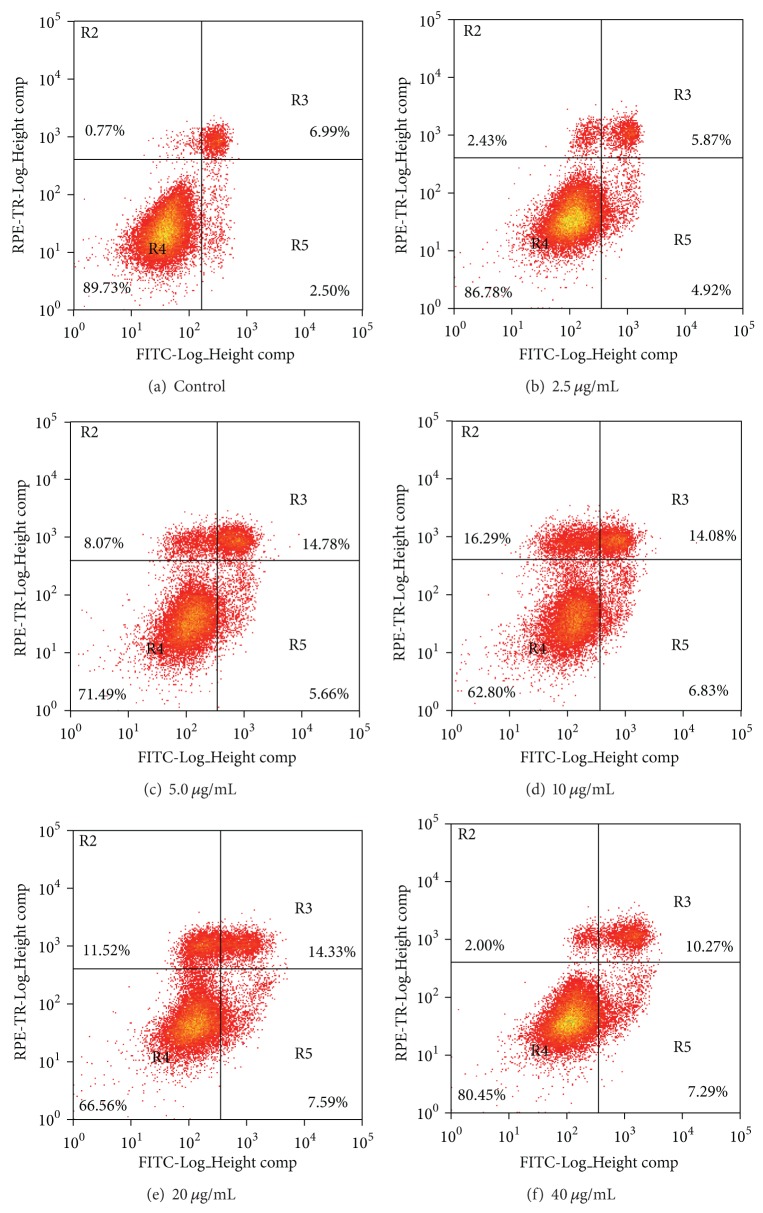
Apoptosis of SHEP cells cultured 24 h in different concentrations of N-APP. (a): Control; (b)–(f): N-APP at different concentrations of 2.5, 5, 10, 20, and 40 *μ*g/mL; for 24 h, apoptosis rates were 4.9%, 5.7%, 6.8%, 7.6%, and 7.3%.

**Figure 8:**
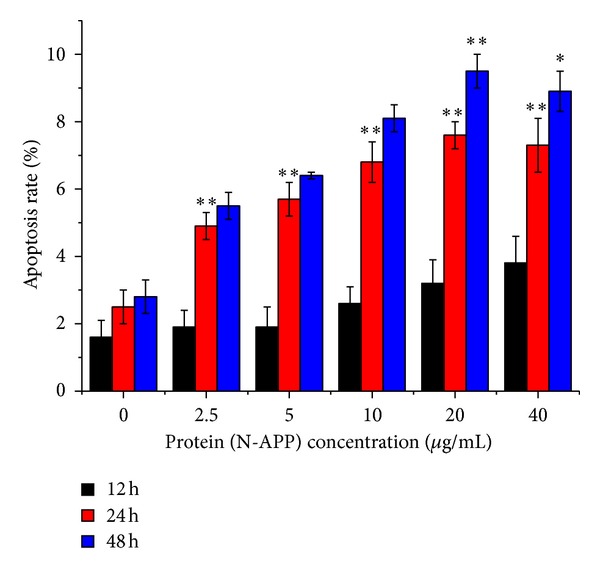
Effect of different concentrations of N-APP on SHEP cells apoptosis with the extension of time.*P < 0.05. **P < 0.01, compared with control group without N-APP. The data were presented as mean ± SD, dealing with different concentrations (0, 2.5, 5, 10, 20, 40 *μ*g/mL) for 12 h, apoptosis rates were (1.6 ± 0.5)%, (1.9 ± 0.5)%, (1.9 ± 0.6)%, (2.6 ± 0.5)%, and (3.2 ± 0.7)%, and (3.8 ± 0.8)%; for 24 h, apoptosis rates were (2.5 ± 0.5)%, (4.9 ± 0.4)%, (5.7 ± 0.5)%, (6.8 ± 0.6)%, (7.6 ± 0.4)%, and (7.3 ± 0.8)%; For 48 h apoptosis rates were (2.8 ± 0.5)%, (5.5 ± 0.4)%, (6.4 ± 0.1)%, (8.1 ± 0.4)%, (9.5 ± 0.5)%, and (8.9 ± 0.6)%.

Effect of N-APP on SHEP cells was further analyzed by flow cytometry. The Annexin V-fluorescein isothiocyante (FITC) kit uses annexin V conjugated with FITC to label phosphatidylserine sites on the membrane surface. The kit includes propidium iodide (PI) to label the cellular DNA in necrotic cells where the cell membrane has been totally compromised. This combination allows the differentiation among early apoptotic cells (annexin V positive, PI negative), necrotic cells (annexin V positive, PI positive), and viable cells (annexin V negative, PI negative). The results showed that N-APP induced cultured cell's apoptosis. The assay not only gives information about the numbers of vital (AV−/PI−) and apoptotic (AV+/PI−) cells, but also concurrently provides the number of necrotic cells (AV+/PI+) (Figure 7). SHEP cells were cultured for 12–48 h in the presence of N-APP with different concentrations. The data were presented as mean ± SD; dealing with different concentrations (0, 2.5, 5, 10, 20, and 40 *μ*g/mL) for 12 h, apoptosis rates were (1.6 ± 0.5)%, (1.9 ± 0.5)%, (1.9 ± 0.6)%, (2.6 ± 0.5)%, (3.2 ± 0.7)%, and (3.8 ± 0.8)%; for 24 h, apoptosis rates were (2.5 ± 0.5)%, (4.9 ± 0.4)%, (5.7 ± 0.5)%, (6.8 ± 0.6)%, (7.6 ± 0.4)%, and (7.3 ± 0.8)%; For 48 h apoptosis rates were (2.8 ± 0.5)%, (5.5 ± 0.4)%, (6.4 ± 0.1)%, (8.1 ± 0.4)%, (9.5 ± 0.5)%, and (8.9 ± 0.6)% (Figure 8). The results showed that the apoptosis rate of the experimental groups was significantly higher than that of the control groups. The cell apoptosis rate was raised with the extension of time. Overall cell apoptosis rate was in upward trend with the increasing concentration of N-APP and only at 40 *μ*g/mL (cultured for 24–48 h) fell slightly. 

## 4. Discussion

Nikolaev et al. [10] reported APP and DR6 activated a caspase-dependent apoptosis program. Their research revealed a new mechanism. APP was cut by a not yet known secretase to yield N-APP in trophic-factor deprivation. The N-APP bound DR6, triggering degeneration of both neuronal cell bodies and axons depend on caspase-3 and caspase-6, respectively. The work also identified a new AD target and proved that the neurodegeneration seen in AD was not only the consequence of accumulate A*β* alone, but a combination of some ignored portions of APP contributing to AD.

In our study, the cDNA encoding N-APP (18-285) with one His-tag and one thrombin-cleavage site was cloned in the* P. pastoris *expression plasmid pPIC9K. His-tag will make the purification procedure easy by Ni+ affinity chromatography; thrombin-cleavage site can cut the His-tag after purification. However, we found it a little difficult for the recombinant protein to bind Ni+ column. Finally, ion exchange chromatography and gel filtration chromatography were used to purify the recombinant protein. Optimum conditions leading to maximum recombinant-protein yield: recombinant strain was cultured in 100 mL BMGY medium 48 h to make full use of the glycerol to get the high density of the cells, then the cell pellets were harvested and resuspended in large volume 200 mL BMMY medium, added 100% methanol to a final concentration of 1% (v/v) at first 24 h, and induction time of 72 h. After purification, the purified protein was very stable with an estimated yield of 50 mg/L. We proved that recombinant N-APP (18-285) induced apoptosis of SHEP cells. The availability of large amounts of high stability purified recombinant proteins could facilitate further studies on its biological activities. Previous studies showed that a cleaved amino-terminal fragment of N-APP could bind DR6 to trigger neuron death via a widespread caspase-dependent self-destruction program, and it was found that DR6 induces apoptosis when it is overexpressed. Data strongly demonstrated that DR6-induced apoptosis is caspase dependent. On the other hand, DR6-induced apoptosis involves cytochrome c release and Bax translocation. DR6 induced apoptosis through a pathway that exclusively depends on the mitochondrial pathway and possibly through interacting with Bax. A conclusion could be drawn that DR6 induces apoptosis of SHEP cells via an intrinsic bax-mitochondrion-cytochrome c-caspase protease pathway. Here, firstly, DR6 could be expressed by SHEP cells as nerve cells, and DR6 is activated locally by N-APP, which triggers neuronal cell body apoptosis because DR6 signals via BAX and caspase 3 in cell bodies. Secondly, the apoptotic rate of SHEP cells as cancer cells is different from normal neuronal cell DR6 is broadly expressed by developing neurons and is required for normal cell body death both *in vivo* and after trophic-factor deprivation *in vitro* [10], but DR6 may be lower expressed in SHEP cells than that of other neurons. So, number of apoptotic cells was found to be very low, even 48 h after addition of high concentration of N-APP, but the cell apoptosis rate was rising with the extension of time. Overall cell apoptosis rate was upward trend with the increasing concentration of N-APP, and only at 40 *μ*g/mL (cultured for 24–48 h) fell slightly. This study was designed to get N-APP in* P. pastoris *and evaluate whether the N-APP could induce apoptosis of SHEP cells. Indeed, its possible mechanisms of N-APP-induced apoptosis in SHEP cells needed to be further studied.

 In conclusion, the work described here provided a feasible method to express high yield recombinant amino-terminal fragment of APP in the methylotrophic yeast *P. pastoris* and established the purification procedure. It would provide a foundation for the further structural and functional characterization of N-APP associated with AD.

## Figures and Tables

**Table 1:** Primer sequence used for PCR amplification of N-APP.

5′ Forward primer 2 *μ*g (312 pmoles),	5′CCG CTCGAG AAAAGA↓CTGGAGGTACCCACTGATGGTAATG3′
lyophilized	*Xho *I Kex2 signal cleavage

3′ Reverse primer	5′GGAATTCCTTATTAATGATGATGATGATGATGGGATCCTCTTGGAACCAACTCTTCCACAGACTCTGT GGTGGTG3′
2 *μ*g (314 pmoles),	*Eco*R I Double stop codon6His-tag Ser Gly ↓ Arg Pro Val Leu
lyophilized	(Thrombin cleavage site)

**Table 2:** Summary of N-APP purification.

Purification step	Total volume (mL)	Total protein (mg)	Purity (%)^a^	Recovery (%)
Culture supernatant	400	204	35	100
Q-Sepharose FF	50	43.1	80	25.9
Ultrafiltration	8	30.4	87	70.5
Sephacryl S-200	13.3	23.6	96	77.6

^a^According to densitometric analysis of SDS-PAGE.
